# Seismic Damage and Behavior Assessment of Drift-Hardening Concrete Walls Reinforced by LBUHS Bars

**DOI:** 10.3390/ma17092070

**Published:** 2024-04-28

**Authors:** Jiayu Che, Bunka Son, Yuping Sun

**Affiliations:** 1 Graduate School of Engineering, Kobe University, 1-1 Rokkodai-cho, Nada, Kobe 657-8501, Japan; 2 Graduate School of System Informatics, Kobe University, 1-1 Rokkodai-cho, Nada, Kobe 657-8501, Japan

**Keywords:** drift-hardening concrete wall, LBUHS bar, seismic behavior, residual drift ratio, maximum strain capacity

## Abstract

This paper experimentally and analytically investigated the damage and seismic behavior of concrete walls reinforced by low-bond ultra-high-strength (LBUHS) bars. To this end, four half-scale rectangular concrete walls were fabricated and tested under reversed cyclic loading and constant axial compression. The test variables were the shear span ratio and the axial load ratio. Based on the test results, the propagation of cracks on the wall surface, the maximum strain capacity of concrete, the hysteresis loops and envelope curves, the residual drifts, and the strain distributions of LBUHS rebars were presented and discussed. The experimental results showed that all the test walls could exhibit drift-hardening capability until at least a 2.0% drift ratio if LBUHS rebars were anchored by nuts at their ends. The test results also indicated that the maximum strain capacity of concrete was above 0.86%, much larger than the currently recommended 0.4%. After unloading from the transient drift ratios of 2.0% and 2.5% for the walls with shear span ratios of 1.5 and 2.0, respectively, the measured residual drift ratios were controlled below 0.4%, which is less than the critical drift ratio (0.5%) having 98% repairable probability recommended in the FEMA document (P-58) for general concrete structures. Furthermore, a numerical method was presented to evaluate the cyclic response of the test walls, and a comparison between the experimental and the calculated results verified the reliability and accuracy of the proposed numerical method.

## 1. Introduction

The fundamental design principles of modern reinforced concrete (RC) structures located in earthquake-prone regions have been to ensure that the structures can withstand “minor tremors without damage, moderate tremors with repairability, and major tremors without collapse”. Following these principles, ductile concrete (DC) walls have been widely used in middle- and high-rise buildings in areas with high seismic risk. In the last several decades, many researchers [1,2,3,4,5,6] have extensively investigated the seismic performance of DC walls and developed ultimate capacity as well as performance-based design methods, which have been recommended in representative seismic design codes [7,8,9,10,11,12,13].

As the primary earthquake-resistant structural components, DC walls and columns have two characteristics, sufficient ductility and sound energy dissipation capacity. However, for conventional ductile concrete structures, both ductility and energy dissipation can only be achieved by permitting damages and residual deformation in the walls and columns. According to the studies and post-earthquake surveys of recent strong earthquakes [14,15,16,17,18,19,20,21,22,23,24,25], the DC walls and columns complying with current design codes performed satisfactorily well under the design basis (DB) earthquakes and could prevent concrete buildings from collapsing. However, some of them sustained severe damage and were demolished [20,21]. From the viewpoint of quick post-earthquake re-occupancy, DC walls are not a panacea for buildings located in earthquake-prone regions. Novel solutions that can achieve higher earthquake-resistant goals than simple life safety [26] need to be developed. 

**Figure 1:**
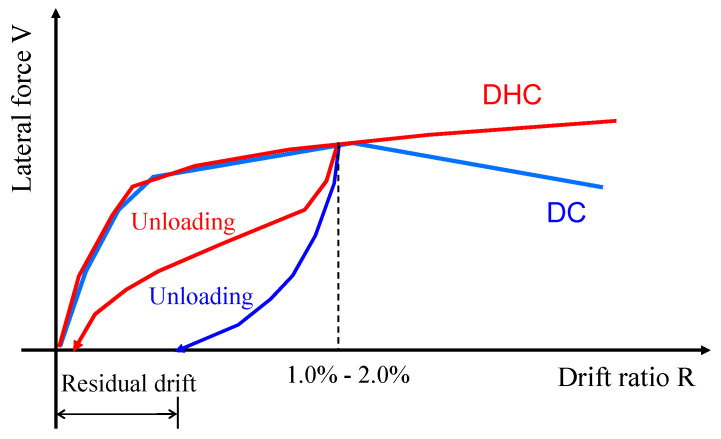
Comparison of seismic performance of DHC and DC structures and/or components.

A drift-hardening concrete (DHC) wall is one candidate for a novel solution. From the comparison in Figure 1, one can see two structural advantages of DHC walls over conventional DC walls: (1) the lateral resistance tends to stably increase with the drift until large deformation, and (2) the residual drift of DHC walls can be reduced significantly. There are two methods for realizing DHC walls. One uses an unbonded prestressed tendon (UPT) to make rocking walls, and the other utilizes LBUHS bars as the longitudinal rebars in the boundary elements of wall panels.

Priestley et al. [27,28] initiated the studies of the rocking walls in the 1990s. Since then, numerous studies have been conducted [29,30,31,32,33,34,35,36] to investigate the seismic behavior of rocking wall systems. On one hand, past investigations have revealed that a rocking wall could restore a building to its original position without residual drift under the DB earthquakes, and design and numerical approaches for selecting the UPT and energy devices have been developed [37,38]. On the other hand, UPTs of rocking walls might break when a structure with rocking walls is shaken by an earthquake stronger than the DB earthquake [33]. This test [33] implies that the rocking wall system should be carefully designed when used in concrete buildings constructed in an area where a stronger earthquake than the DB earthquake is expected. 

To more effectively and simply make DHC columns and walls, the last author and his colleagues [39] have proposed utilizing SBPDN bars as longitudinal rebars of concrete columns and walls. The SBPDN bar is one of the LBUHS bars with spiraled grooves on its surface. The yield strength of the SBPDN bar is 1275 MPa, but its bond strength is much lower than that of a deformed bar [40]. The last author and his research team have clarified that the use of SBPDN rebars could ensure a drift-hardening capability of 4.0% drift and larger to concrete columns [41,42] and demonstrated that the larger elasticity and low bond strength of SBPDN rebars could prevent the tensile reinforcement in concrete components from early yielding and enable concrete columns to return to their original positions after being deformed to large deformation. 

Though there are several studies on the seismic behavior of concrete walls reinforced by PC strands [43] and by CFPR bars [44], there is little, if any, information on the seismic properties of the DHC walls reinforced by LBUHS rebars, which have much higher compression-resistant capacity than PC strands and CFRP bars and are expected to ensure that concrete walls have a larger drift-hardening capability.

In analyzing the structural performance of reinforced concrete components, researchers and engineers have mainly used general-purpose FEM software packages such as ABAQUS 2022, ANSYS 2024 R1, DIANA 10, and OpenSEES 3.6.0 around the world [45,46,47,48].

On the other hand, due to their low bond strength, SBPDN rebars tend to slip along with the drift, and it is still a challenge for current software to reliably simulate the cyclic response of DHC walls reinforced by SBPDN rebars. To promote the application of DHC walls in building structures, the development of a reliable and accurate analytical method that can consider the effect of slippage of the SBPDN rebars is also indispensable.

This paper aimed (1) to provide experimental information on the seismic performance of DHC walls reinforced by SBPDN rebars, (2) to provide experimental information on the propagation of flexural and shear cracks and the maximum strain capacity of concrete, (3) to verify the reliability of the anchorage of SBPDN rebars by nuts and washers through cyclic loading testing of DHC walls, (4) to investigate the influence of the shear span ratio and the axial load level on the seismic damage and behavior of the DHC walls, and (5) to propose a numerical method that can take account of bond slippage of SBPDN rebars for predicting the hysteresis behavior of DHC walls and confirm its accuracy.

## 2. Experimental Program

### 2.1. Outlines of the Specimens and Material Properties

**Figure 2:**
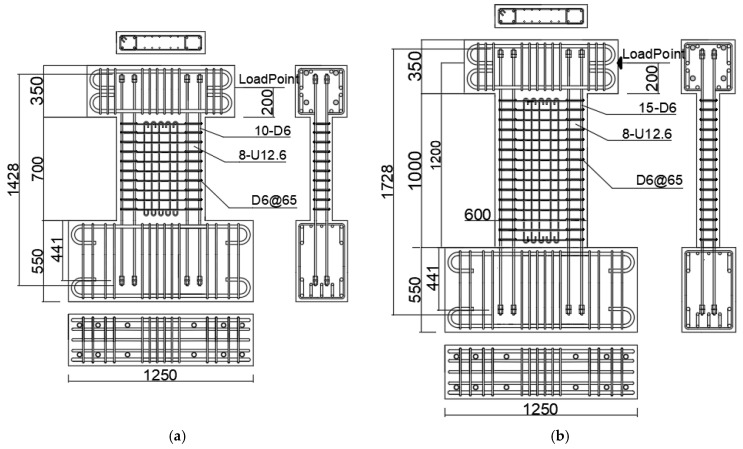
Dimensions and reinforcement details: (**a**) W15-075 and W15-150; (**b**) W20-075 and W20-150.

To achieve the research goals mentioned above, four 1/2 scale specimens were made. All the specimens were tested under reversed cyclic lateral loading and constant axial load. Table 1 lists experimental parameters along with the primary test results of the test walls, and Figure 2 shows reinforcement details and dimensions. 

**Figure 3:**
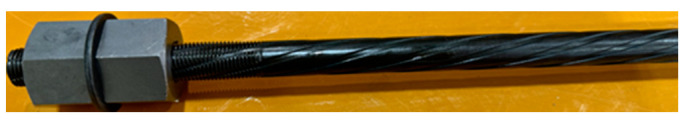
The surface of an LBUHS bar and mechanical anchorage by washer and nuts.

As shown in Figure 2, the section of all the test walls was 150 mm × 600 mm. The heights of specimens, measured from the wall bottom to the action line of lateral load, were 900 mm and 1200 mm for the specimens with shear span ratio (a/D ratio) of 1.5 and 2.0, respectively. Experimental variables were the a/D ratio (1.5 and 2.0) and the axial load ratio n (=*N*/(*Dtf_c_^’^*)). As one can see from Figure 2, the longitudinal rebars placed in each edge zone of the wall panel comprised four SBPDN rebars (U12.6) having a nominal diameter of 12.6 mm (see Figure 3). The ends of U12.6 rebars were fixed via nuts to a washer. The numeral 441 in Figure 2 expresses the embedded length, measured from the center of the washer to the bottom of the wall panel in mm, and is 35 times the nominal diameter of U12.6 rebar. 

The horizontal reinforcement in the wall panel was composed of rectangular hoops made of D6 deformed bars with a spacing of 65 mm to give a transverse steel ratio of 0.65%, while the longitudinal reinforcement consisted of ten D6 deformed bars with a spacing of 65 mm as shown in Figure 2. Table 2 shows the mechanical properties of the U12.6 bar and D6 deformed bar.

Portland cement and coarse aggregates with a maximum size of 20 mm were used to make the specimens. The compressive strengths of 100 mm × 200 mm concrete cylinders at the testing stages are shown in Table 1. The cylinders were cured under the same conditions as the specimens.

### 2.2. Loading Apparatus and Instrumentations

**Figure 4:**
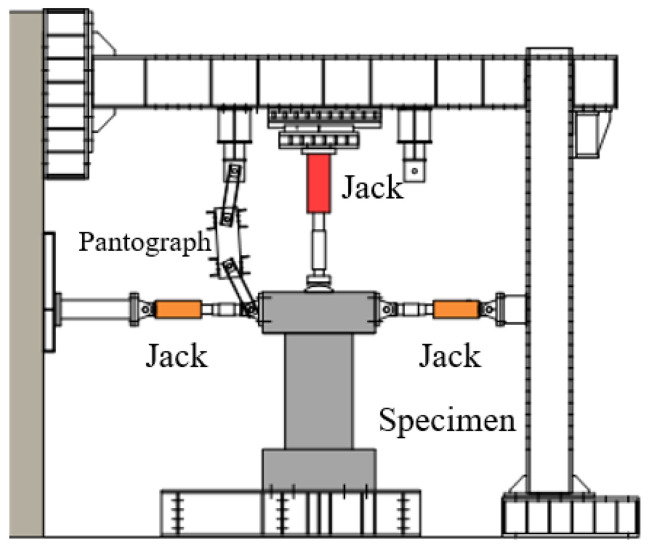
Loading apparatus.

**Figure 5:**
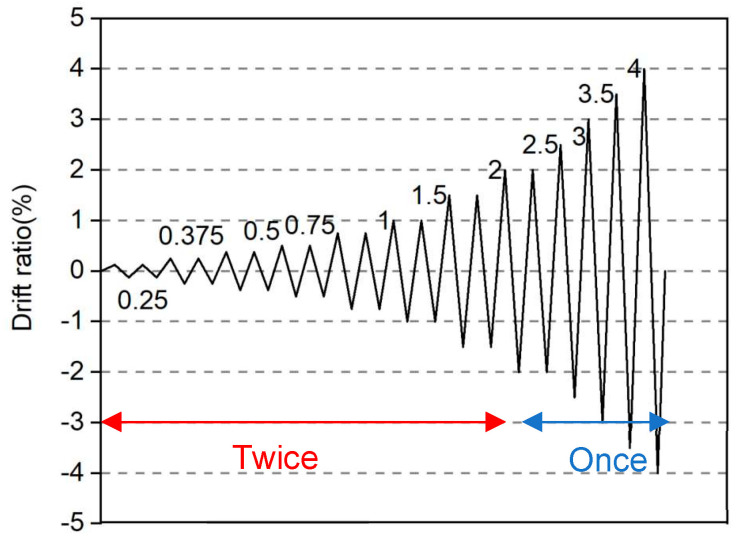
Loading program.

Figure 4 illustrates the test setup. The axial load was applied using a 1 MN capacity hydraulic jack before the cyclic lateral loading was applied. Two jacks with 300 kN capacity in pull were used to apply the cyclic lateral load. A cylindrical steel seat was set between the upper loading beam and the 1 MN jack to maintain the rotation center on the action line of the reversed lateral load. The cyclic lateral force was controlled by the drift ratio (R), which is the ratio of the tip lateral displacement to the shear span of the wall. The loading program, which has been generally adopted in Japan, is shown in Figure 5.

As one can see from Figure 5, displacement control was applied during the entire loading, and the lateral load was applied for two complete cycles at the drift ratios of 0.125%, 0.25%, 0.375%, 0.5%, 0.75%, 1.0%, 1.5%, and 2.0%. Only one complete cycle was performed at the drift ratios of 2.5%, 3%, 3.5%, and 4%. The testing was terminated either when the test wall could not sustain axial load anymore or when the lateral load degraded below 85% of the peak load.

**Figure 6:**
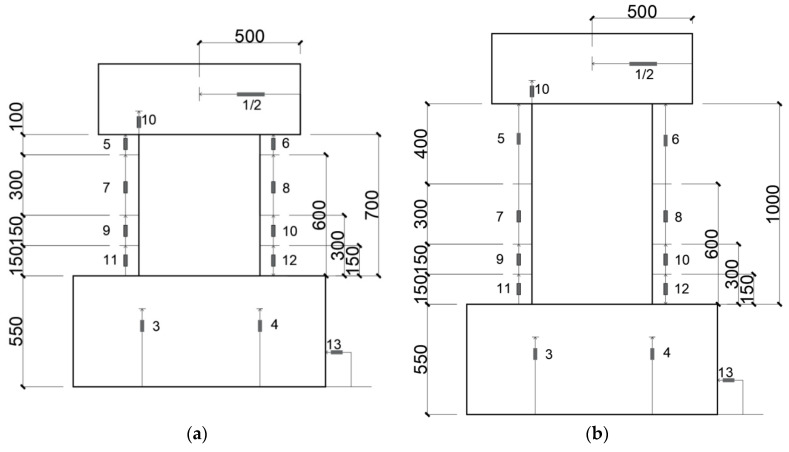
Locations of displacement transducers (DTs): (**a**) specimens with a/D = 1.5; (**b**) specimens with a/D = 2.0.

Figure 6 displays the locations of the displacement transducers (DTs). Thirteen DTs were mounted to measure the lateral and vertical displacement. As is obvious from Figure 6, DTs 1 and 2 were used to measure the tip lateral displacement, and DTs 3 and 4 were used to monitor the rotation of the bottom loading beam. The overall vertical deformation of the specimens was measured by DTs 5 and 6. The displacement traducers DTs 7 through 12 were aimed to measure the axial deformation with gauge lengths of 150 mm, 300 mm, and 600 mm. These gauge lengths are the distances from the upper surface of the bottom loading beam. The potential slip of the lower loading beam was monitored by DT 13.

**Figure 7:**
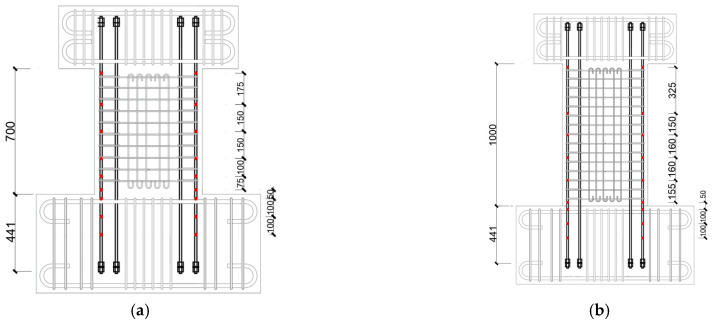
Locations of strain gauges: (**a**) specimens with a/D = 1.5; (**b**) specimens with a/D = 2.0.

To measure the axial strains of SBPDN rebars, a total of eighteen uniaxial strain gauges (self-temperature-compensation from 10 to 100 °C) were embedded on the surfaces of the SBPDN rebars at the outermost tension and compression sides. Positions of the strain gauges are shown in Figure 7 by red points.

## 3. Experimental Results and Discussion

### 3.1. Damage and Crack Propagation

**Figure 8:**
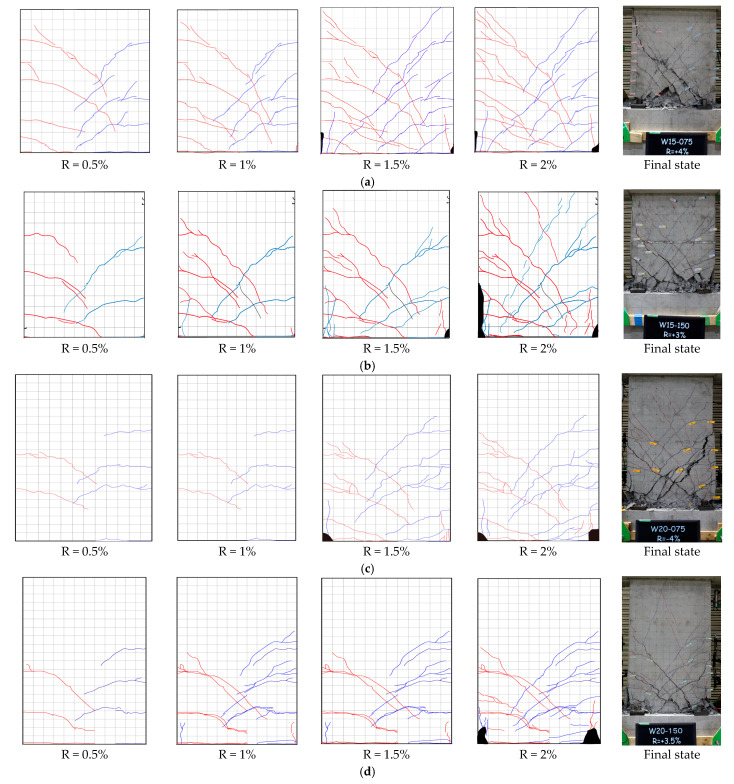
Propagation of cracks observed: (**a**) W15−075; (**b**) W15−150; (**c**) W20−075; (**d**) W20−150.

Figure 8 displays the crack patterns observed on the wall panel at the transient drift ratios of 0.5%, 1.0%, 1.5%, and 2.0% along with the photos that were taken after the testing and show the final states of all specimens. In Figure 8, the grid size on the surface of the wall panel is 50 mm. The red lines represent the cracks observed in the push direction, while the blue lines express the cracks observed in the pull direction. In addition, the black areas near the wall toes represent the area of spalling-off concrete. 

As is obvious from Figure 8, regardless of the shear span ratio, most of the cracks were concentrated in the end region (about 1.0 D) from the wall toes, and all the specimens exhibited flexure-dominated crack patterns until the drift ratios of 1.5%. Pulling out of SBPDN rebars was not observed in any test wall until the end of testing. 

The first flexural cracks occurred in specimen W15-075 around the drift ratio of 0.125%, and the flexural cracks gradually propagated into diagonal shear cracks at R = 0.25%. When R approached 1.0%, flaking of the cover concrete was observed at the compressive edge of the wall panel, but obvious spalling off of the compressed concrete did not occur until R = 1.5%. The specimen reached its maximum lateral force at R = 2.0%, after which the shear cracks widened and the lateral resistance decreased as the drift ratio increased. The specimen failed in shear while pushing towards the drift ratio of 4.0%.

For specimen W15-150, flexural cracks were first observed at the drift ratio of 0.125%. The cover concrete commenced flaking near R = 1.0% and began to spall near R = 1.5%. The specimen reached maximum lateral load at R = 2.0%. From that drift ratio on, the crushing of concrete became more and more severe due to the higher compression, resulting in sharper degradation of lateral resistance than that for specimen W15-075. The specimen failed in shear during pushing towards R = 3.0%.

As for specimen W20-075, the first flexural crack was confirmed at R = 0.125% and started propagating diagonally when the drift ratio approached 0.25%. The cover concrete flaked near R = 0.75% and R = −1.0% in the push and pull directions, respectively. The compressed concrete spalled off at the drift ratio of 1.5%. The specimen reached the maximum lateral resistance at R = ±3.0%. Severe crushing of concrete near the wall toes caused failure of the specimen at R = −4.0%.

For specimen W20-150, the first flexural cracks occurred around R = 0.125%. As in other specimens, the flexural cracks commenced propagating diagonally near R = 0.25%. The concrete shell at the compression side flaked at the wall bottom when R reached ±1%. Spalling off of the compressed concrete was observed at the drift ratios of ±1.5%. After reaching the maximum lateral resistance at R = 3.0% (R = −2.5%), the specimen failed at R = −3.0% because the compressive concrete sustained severe damage.

**Figure 9:**
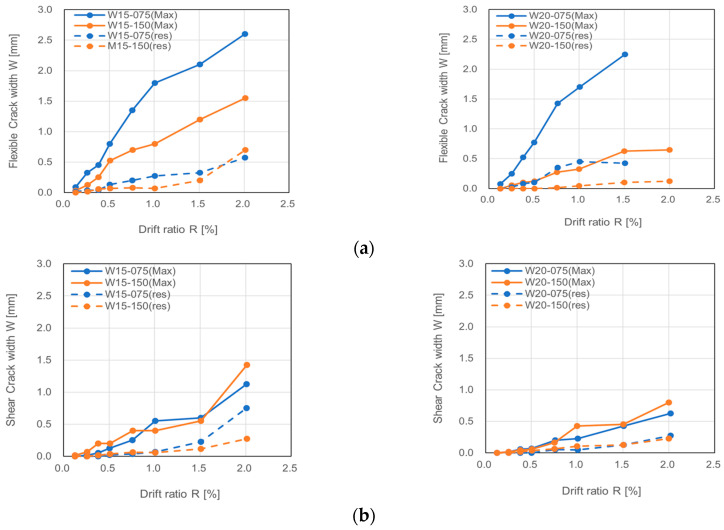
Maximum and residual widths of cracks at transient drift ratios: (**a**) flexural crack; (**b**) shear crack.

To better show the propagation process of cracks, Figure 9 shows the maximum and residual widths of the flexural and shear cracks measured at each transient drift ratio. The solid curves and dotted ones represent the maximum width and residual width of cracks, respectively. The crack widths shown in Figure 9 express the average of the crack widths measured in both directions. The measured crack widths are shown only until R = 2.0% because after that drift ratio the measured crack widths become unreliable due to the influence of the severe spalling off of concrete.

From Figure 9, one can see that the larger the axial load ratio and the shear span ratio, the less the maximum width of a flexural crack. The maximum width of a shear crack was subjected to little, if any, influence by the axial load level but did decrease as the shear span ratio increased. It is noteworthy that the residual width of a flexural crack even after unloading from the drift ratio of 2.0% was less than 1.0 mm, which is the upper limit corresponding to the minor damage state prescribed in the AIJ design guideline [45], implying that the use of LBUHS rebar is effective in reducing the damage degree of concrete walls. It is further noteworthy that the residual width of a shear crack after unloading from R = 1.5% was less than 0.3 mm, below which repairing is not needed and the reoccupation of buildings is permissible [49]. 

### 3.2. Lateral Force versus Drift Ratio Relationships

**Figure 10:**
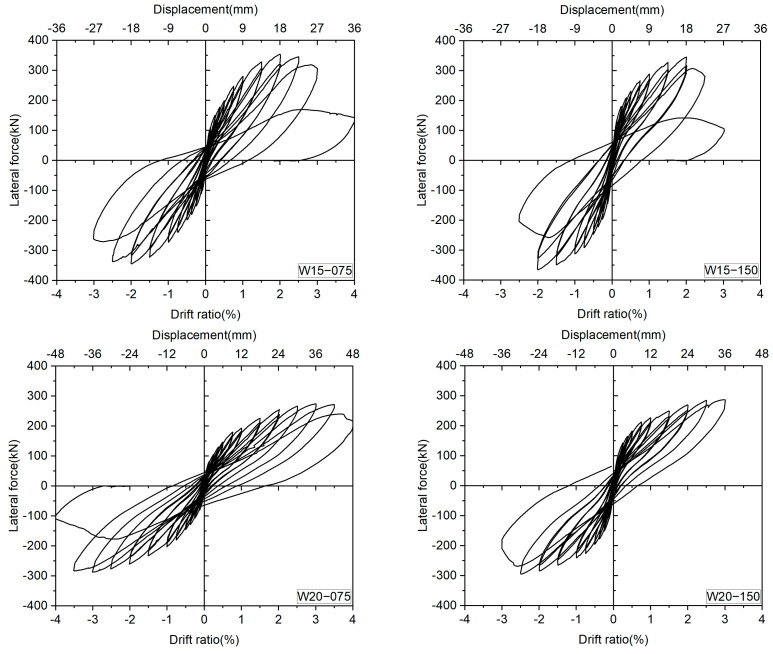
Measured lateral force versus drift ratio relationships.

The measured lateral force versus drift ratio relationships are shown in Figure 10. As displayed in Figure 10, the test walls all exhibited typical flexure-dominant hysteresis loops and clear drift-hardening capability up to the drift ratio of at least 2.0%. The peak drift ratios increased with the shear span ratio, while an increase in the axial load level led to a decrease in the peak drift ratio. Shear failure occurred in the specimens with a/D = 1.5 under the axial load ratio of 0.075 and 0.15 at R = 3.0% and 2.5%, respectively, while specimens with a/D = 2.0 failed due to the severe crushing of concrete at the drift levels of 4.0% and 3.0%, respectively. 

**Figure 11:**
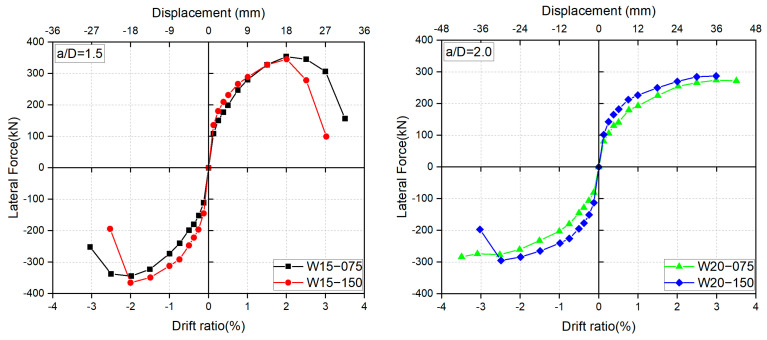
Influence of the experimental variables.

To present the influence of the experimental variables on the overall seismic behavior of the DHC walls, the skeleton curves of all specimens are depicted in Figure 11. One can see from Figure 11 that the axial load ratio affects not only the ultimate lateral resistance of the DHC walls, but also the initial stiffness and deformability. Generally, the higher the axial load level, the larger the initial stiffness and the ultimate lateral resistance, but the lower the peak drift ratios and the sharper the degradation of lateral resistance after the peaks.

### 3.3. Measured Maximum Strain Capacity of Compressed Concrete

**Figure 12:**
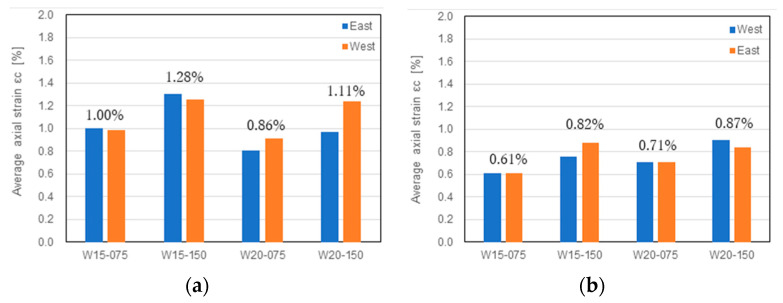
Measured maximum strain capacities of concrete: (**a**) GL = 150 mm; (**b**) GL = 300 mm.

Maximum strain capacity is an important index measuring the damage state of compressed concrete, and it is generally defined as the strain at which concrete loses its compressive resistance [50]. Figure 12 displays the experimental maximum strain capacities of concrete measured at ±1.5% drift ratios, at which the cover concrete clearly spalled off. The experimental strain capacities were obtained by dividing the vertical displacements measured via DTs 11 and 12 (see Figure 6) by the gauge length of 150 mm. The legend entries “east” and “west” in Figure 11 express the compressive strains measured at the east and the west edges of the wall panel, respectively. To be more specific, the “east” and “west” strains shown in Figure 12 correspond to the axial strain measured at R = 1.5% and R = −1.5%, respectively. Furthermore, since the spalling off of cover concrete at R = ±1.5% was concentrated in the 150 mm end portion of the specimens (see Figure 8), it is rational and acceptable to adopt the average axial strain within the end region with the gauge length (GL) of 150 mm as the maximum strain capacity of concrete. For reference, the maximum strain capacities measured with the end region with GL = 300 mm are also plotted in Figure 12.

As shown in Figure 12, the smallest average maximum strain capacity reached 0.86%, much larger than the strain of 0.4% currently recommended for concrete [46]. Because the strain of 0.4% is based on the tests of concrete specimens under concentric loading, the much larger maximum strain capacities shown in Figure 12 can be attributed to the effects of the strain gradient across the wall section and of the moment gradient along the wall height. Both gradients may provide beneficial effects to the extremely compressed concrete and may enhance its maximum strain capacity [51]. One also can see from Figure 12 that the shorter the shear span ratio, the larger the maximum strain capacity, because a shorter shear span ratio means a larger moment gradient.

### 3.4. Residual Deformation

Residual deformation is an indicator used to evaluate the reparability and restorability of concrete structures. McCormick et al. [52] proposed the use of residual deformation to define the limit states in performance-based seismic design frameworks and specifically suggested 0.5% as an allowable residual drift ratio. This 0.5% residual drift ratio has also been recommended as the critical residual drift ratio having a 98% repairable probability by FEMA [53]. 

**Figure 13:**
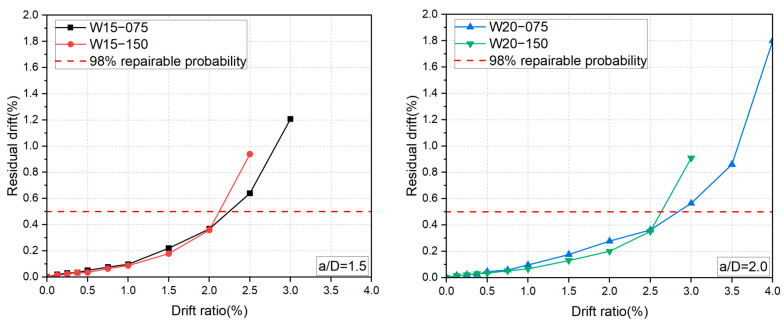
Measured residual drift ratios.

To clarify the high reparability of the DHC walls, Figure 13 summarizes the measured residual drift ratios. As is apparent from Figure 13, the residual drift ratios decreased as the shear span ratio increased. The residual drift ratios corresponding to the 2.0% and/or 2.5% transient drift ratios were all kept at less than 0.4%. The axial load level had little, if any, influence on the residual drift ratio. These observations imply that the utilization of SBPDN rebars is very effective in reducing the residual drift of concrete walls and enhancing their reparability.

### 3.5. Strain History of LBUHS Rebars

**Figure 14:**
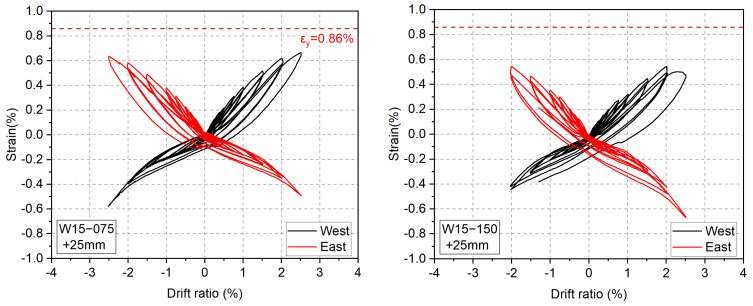

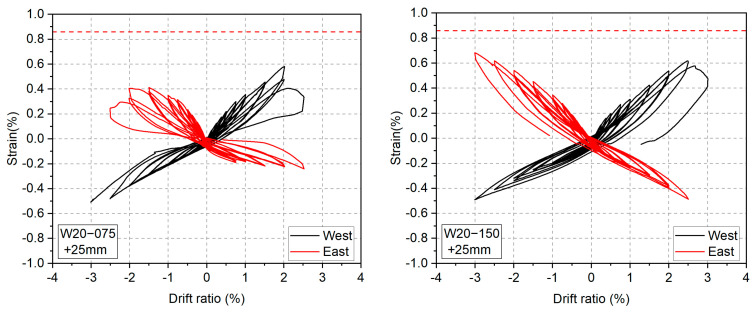
Strain hysteresis of SBPND rebars.

Figure 14 shows the experimental results of the axial strains of SBPDN rebars. The strains shown in Figure 14 represent those located at the section 25 mm away from the wall bottom. As can be seen from Figure 14, for all specimens, SBPDN rebars did not yield until the peak drift ratios. The low bond resistance did delay the yielding of SBPDN rebars, which allows for a stable increase in the stress and the lateral resistance of SBPDN rebars with the drift ratio, providing the walls with sufficient drift-hardening capability up to large drift ratios.

## 4. Numerical Analytical Method for Assessing Seismic Behavior of DHC Walls

### 4.1. Outlines of Numerical Analytical Method

Because SBPDN bars embedded in concrete with a strength of 40.0 MPa have a bond strength of about 3.0 Mpa, only one-fifth of that of deformed high-strength bars [40], it is conceivable that SBPDN rebars will slip with drift ratio and affect the overall seismic performance of the DHC walls. Therefore, to reliably and accurately assess the cyclic behavior of the DHC walls, it is indispensable to take the effect of slippage of SBPDN rebars into consideration in the numerical analysis.

**Figure 15:**
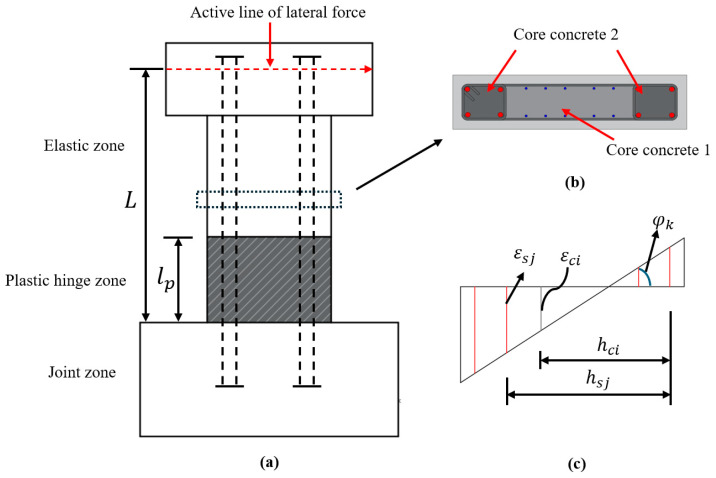
Discretization of the wall along the height and within the section: (**a**) elevation; (**b**) cross section of wall section; (**c**) strain profile across the wall section.

The finite spring method (FSM) has been developed by Sun et al. [54] to assess the cyclic behavior of concrete components. In the FSM, concrete columns and walls are discretized into joint segments, plastic hinge segments, and elastic segments (see Figure 15). To see the effect of bond slippage of longitudinal rebars, the joint and elastic segments are further divided into finite elements, each of which is replaced by a bond-slip spring having a reliable bond stress–slip relation. The FSM will be adopted in this paper to assess the hysteresis behavior of the specimens.

Four basic assumptions were made in conducting numerical analysis by FSM. They are as follows: (1) the tensile resistance of concrete can be ignored, (2) the plane-remain-plane assumption exists only for the concrete section, (3) the stress–strain relations of concrete and longitudinal rebars are known, and (4) the lateral displacement is mainly due to the flexural deformation in the plastic hinge regions, with the lengths of 0.65 D and 0.75 D (D is the section depth) for the specimens with a/D = 1.5 and 2.0, respectively [55]. 

**Figure 16:**
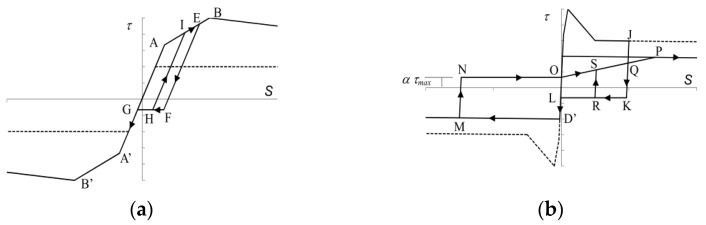
Bond stress–slip relationship of SBPDN rebars: (**a**) before the peak; (**b**) after the peak.

The stress–strain models proposed by Sun et al. [56] and Menegotto et al. [57] will be adopted for concrete and SBPND rebars, respectively. Details of these models can be found in respective references. The Funato model [40] will be applied to define the bond stress–slip relationship of SBPDN rebars. The envelope curve as well as the unloading and reloading rules are shown in Figure 16. The critical parameters defining the complete bond stress–slip models were based on the pulling-out tests of SPBDN bars. The maximum bond stress (bond strength, *τ_max_*) and slip are taken as 3.0 Mpa and 0.015 mm, respectively, and the residual bond stress is assumed to be 0.13*τ_max_* [40]. 

The procedures of numerical analysis of the hysteresis behavior of DHC walls have been described in the work by Sun et al. [54] in detail.

### 4.2. Comparison between the Measured and the Calculated Results

**Figure 17:**
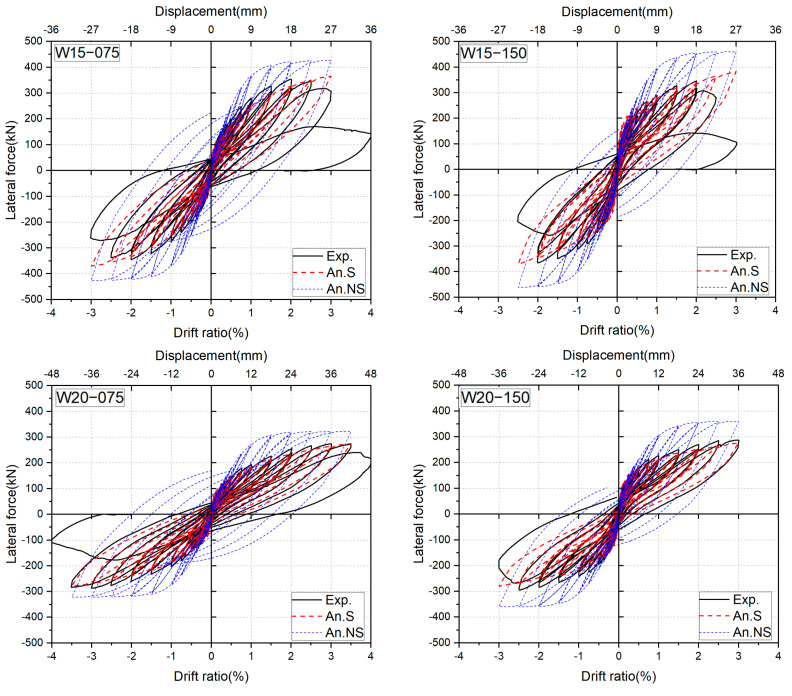
Comparisons of experimental and analytical hysteresis loops.

**Figure 18:**
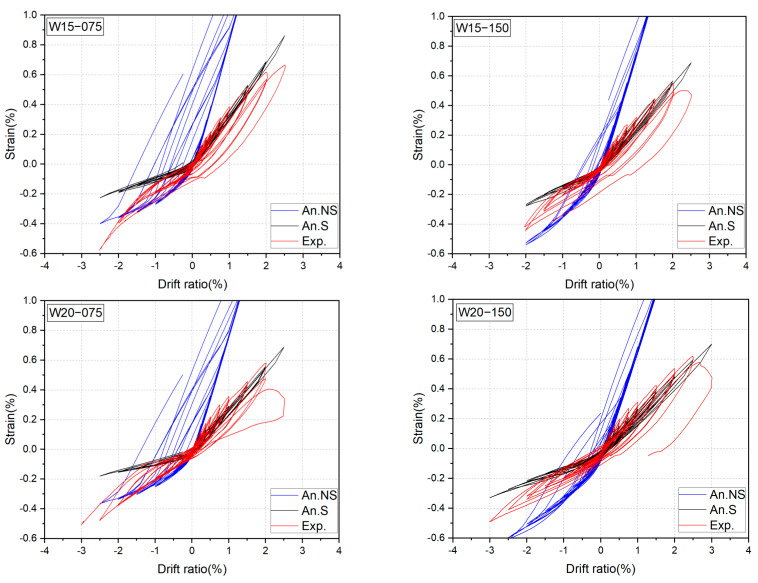
Comparisons of experimental and analytical strains of SBPDN rebars.

**Figure 19:**
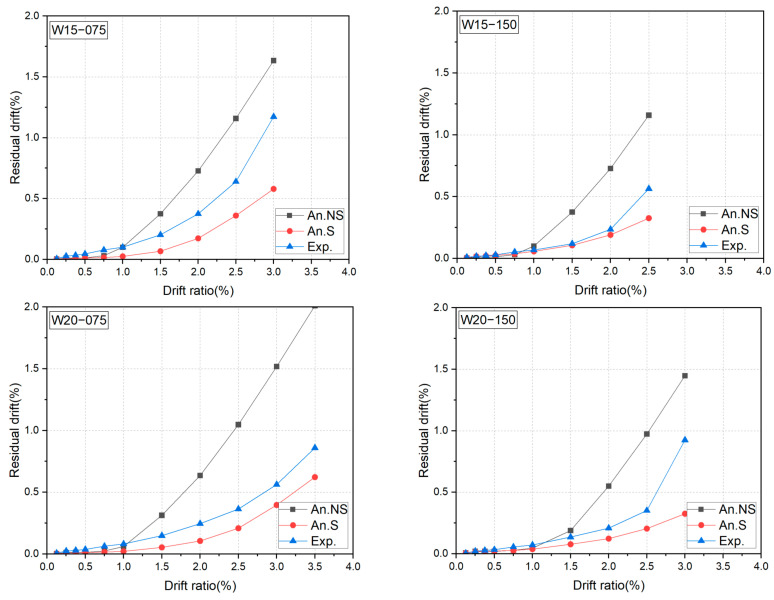
Comparisons between tested and analytical residual drift ratios.

To verify the reliability and accuracy of the proposed numerical method, the calculated results by the FSM will be compared with the experimental results in the key aspects inclusive of the hysteresis loop, the strain history of longitudinal bars, and the residual drift ratio. The comparisons are shown in Figure 17, Figure 18 and Figure 19, where the calculated results considering the effect of bond slippage are labeled as “An.S” and expressed in red dashed lines. For comparison, the calculated results ignoring the bond slippage effect are superimposed in these figures in blue dotted lines and labeled as “An.NS”.

It can be seen from Figure 17 that the numerical hysteresis loops considering the effect of the bond slippage could predict the measured cyclic behavior up to the peak points with very satisfactory accuracy, and the calculated peak loads were only 4–7% lower than the measured ones. However, the calculated results without considering the effect of bond slippage overestimated the peak lateral resistances by 11–21%. The importance of the consideration of the bond slippage can also be seen from the comparisons shown in Figure 18. 

As is apparent from Figure 18, the calculated strains could trace the strain histories of SBPDN rebars up to the peak drift ratios, while the ignorance of the bond slippage effect might lead to an overestimation of the steel strain from the small drift level of 0.25% on, resulting in the overestimation of the lateral resistance of DHC walls shown in Figure 17. 

As for the residual drift ratios, one can see from Figure 19 that the calculated residual drift ratios considering the bond slippage effect tended to underestimate the measured results, while ignorance of the bond slippage effect gave an unconservative prediction of residual drift ratios. The main reason for the discrepancy between the measured and the calculated residual drift ratios is that the FSM cannot quantitatively trace the crushing process of concrete, nor accurately consider the effect of concrete crushing. To enhance the accuracy and widen the applicability of the FSM, further modification is needed to cover the influence of the crushing of concrete and the shear degradation at large drifts. 

## 5. Conclusions

To verify the effectiveness of an end-anchorage method for LBUHS rebars in realizing drift-hardening concrete (DHC) walls, four concrete walls reinforced by SBPDN rebars were made and tested under reversed cyclic lateral loading and constant axial compression. The influences of the axial load ratio and shear span ratio on the seismic damage and overall behaviors of the DHC walls were investigated. The main findings obtained from the test results and numerical analysis described in this paper can be summarized as follows:

1. The use of SBPDN bars as longitudinal rebars, which were mechanically anchored by nuts and washers and had enough embedded length, could ensure the drift-hardening capability of concrete walls up to the drift ratio of at least 2.0%, which is the collapse prevention drift ratio recommended in FEMA 356 [58] for ductile concrete walls.
2. The peak drift ratios of the DHC walls reinforced with SBPDN rebars increased as the axial load level decreased and the shear span ratio increased. For the specimens under an axial load ratio of 0.075, the DHC walls with a/D ratios of 1.5 and 2.0 exhibited drift-hardening behavior until 2.5% and 3.0% drift ratios, respectively. As the axial load ratio increased to 0.15, the DHC walls still maintained drift-hardening behavior up to 2.5% and 3.0% drift ratios, respectively.
3. The larger the axial load ratio and the shear span ratio, the less the maximum width of the flexural crack. The maximum width of the shear crack was subjected to little, if any, influence by the axial load level but decreased as the shear span ratio increased. The residual width of a flexural crack even after unloading from R = 2.0% was less than 1.0 mm, the upper limit for the minor damage state in the AIJ design guideline, implying that the use of SBPDN rebars is effective in reducing the damage degree of concrete walls. It is further noteworthy that the residual width of a shear crack after unloading from R = 1.5% was less than 0.3 mm, below which no repairing is needed and the reoccupation of buildings is permissible.
4. The smallest average maximum strain capacity reached 0.86%, much larger than the strain of 0.4% widely recommended for concrete because of strain gradient and moment gradient effects, both of which may provide the compressed concrete with extra confinement, enhancing its maximum strain capacity.
5. The residual drift ratios decreased as the shear span ratio increased, and they were kept below 0.4% after being unloaded from the 2.0% and 2.5% transient drift ratios for the DHC walls with shear span ratios of 1.5 and 2.0, respectively, regardless of the axial load ratio. The influence of the axial load ratio on the residual drift ratio was little, if any, until the peaks of the DHC walls.
6. The presented FSM could trace the hysteresis loops of the DHC walls up to the drift ratios of at least 2.0% with very satisfactory accuracy. On the other hand, ignoring the bond slippage effect might result in an unconservative prediction of the experimental loops from the 0.5% drift ratio on and overestimate the peak lateral resistances by 11–20%. To trace the post-peak performance and to more accurately predict the residual drift ratio, the influence of the shear failure at large deformation and the crushing of concrete need to be taken into consideration.

## Figures and Tables

**Table 1:** Experimental parameters and main test results.

Specimens	*f_c_’* (MPa)	*a/D*	*n*	LBUHS Rebars	Longitudinal Bars		Horizontal Bars	
					*ρ_w__v_* (%)	Type	*ρ_wh_* (%)	Type
W15-075	45.6	1.5	0.075	4-U12.6	0.35	10-D6	0.65	Hoops D6@65
W15-150	43.7	1.5	0.15					
W20-075	45.1	2.0	0.075					
W20-150	44.5	2.0	0.15					

*f_c_’*: strength of concrete cylinder, *n*: axial load ratio (=*N/tDf_c_*; *N*: axial load, *t*: thickness of wall section), *ρ_wv_*: steel ratio of longitudinal reinforcement, *ρ_wh_*: steel ratio of transverse reinforcement.

**Table 2:** Mechanical properties of the rebars used.

Notation		*E_s_*	*f_sy_*	*e_y_*	*f_su_*
		(kN/mm^2^)	(N/mm^2^)	(%)	(N/mm^2^)
D6	SD295A	196	400	0.23	530
U12.6	LBUHS	212	1399 *	0.86 *	1480

*E_s_*: Young’s modulus, *f_sy_* and *e_y_*: yield strength and strain, *f_su_*: tensile strength. * 0.2% offset yield strength and strain.

## Data Availability

The data are contained within the article.
